# Biologically informed variational autoencoders allow predictive modeling of genetic and drug-induced perturbations

**DOI:** 10.1093/bioinformatics/btad387

**Published:** 2023-06-16

**Authors:** Daria Doncevic, Carl Herrmann

**Affiliations:** Health Data Science Unit and BioQuant, Medical Faculty Heidelberg, Im Neuenheimer Feld 267, 69120 Heidelberg, Germany; Health Data Science Unit and BioQuant, Medical Faculty Heidelberg, Im Neuenheimer Feld 267, 69120 Heidelberg, Germany

## Abstract

**Motivation:**

Variational autoencoders (VAEs) have rapidly increased in popularity in biological applications and have already successfully been used on many omic datasets. Their latent space provides a low-dimensional representation of input data, and VAEs have been applied, e.g. for clustering of single-cell transcriptomic data. However, due to their non-linear nature, the patterns that VAEs learn in the latent space remain obscure. Hence, the lower-dimensional data embedding cannot directly be related to input features.

**Results:**

To shed light on the inner workings of VAE and enable direct interpretability of the model through its structure, we designed a novel VAE, OntoVAE (Ontology guided VAE) that can incorporate any ontology in its latent space and decoder part and, thus, provide pathway or phenotype activities for the ontology terms. In this work, we demonstrate that OntoVAE can be applied in the context of predictive modeling and show its ability to predict the effects of genetic or drug-induced perturbations using different ontologies and both, bulk and single-cell transcriptomic datasets. Finally, we provide a flexible framework, which can be easily adapted to any ontology and dataset.

**Availability and implementation:**

OntoVAE is available as a python package under https://github.com/hdsu-bioquant/onto-vae.

## 1 Introduction

In recent years, deep learning (DL) has been widely used to analyze high-dimensional biological omics data, especially single-cell RNA sequencing (RNA-seq). In contrast to linear methods, such as principal component analysis (PCA), non-linear models can capture more complex patterns in the data (Svensson *et al.* 2020). One prominent example of an unsupervised DL model that performs dimensionality reduction is the autoencoder (AE) (Hinton and Salakhutdinov 2006), which consists of two neural networks: an encoder, which compresses the data, and a decoder, which then aims at reconstructing the input data from this compressed representation that is also referred to as latent space. A more recent variant of the AE is the variational autoencoder (VAE) (Kingma and Welling 2013), which learns a probability distribution over the latent vectors of the data and thus belongs to the class of generative models. AE-based methods have successfully been applied in the context of cancer classification (Zhang *et al.* 2019), data integration (Zhang *et al.* 2019), data denoising (Eraslan *et al.* 2019) and batch correction (Wang *et al.* 2019), cell clustering (Tran *et al.* 2021), multi-domain translation (Yang *et al.* 2021), and prediction of effects of drug treatment on single-cells (Lotfollahi *et al.* 2019). However, in contrast to PCA, these AE-based approaches lack interpretability as we cannot easily assign feature contributions to the latent vectors due to their intrinsically non-linear nature.

Different approaches have already been used to tackle the problem of limited interpretability. Tybalt tries to extract a biologically meaningful latent space by examining how different latent vectors separate covariates and then investigating the associated gene weights of the latent vectors of interest in the one-layer decoder (Way and Greene 2018). Other models modify the decoder to trade off reconstruction accuracy for interpretability. For example, in the LDVAE model, a linear decoder has been implemented to allow assignment of feature weights to the different latent vectors (Lopez *et al.* 2018, Svensson *et al.* 2020). Another approach is the direct modification of the neural network structure through the incorporation of prior biological knowledge. In the VEGA and expiMap models, the authors used a one-layer, sparse decoder that connects the latent variables to a set of annotated genes, thus providing direct interpretability of the latent variables, which can represent different biological entities, such as pathways or transcription factors (Seninge *et al.* 2021, Lotfollahi *et al.* 2023). One limitation of these models is the simplicity of their structure, which does not allow the incorporation of more complex, hierarchical biological information. Other approaches have been aiming at incorporating hierarchical biological networks into a neural network. Knowledge-primed neural networks, in which every node represents a protein or gene, and every edge a regulatory interaction, have been used to model T cell receptor stimulation (Fortelny and Bock 2020). DCell is structured according to subsets of Gene Ontology (GO) and has been used to predict growth rates in yeast and the impact of double-mutants on biological processes (Ma *et al.* 2018). Gene Ontology Autoencoder (GOAE) implements GO terms in one hidden layer of encoder and decoder by partial connectivity to input and output layer, and has been used for clustering of single-cell RNA-seq data, however, as the ontology information is only used in one layer, GOAE can only model relationships between GO terms and genes, but not between parent and children GO terms (Peng *et al.* 2019). Deep GONet is a neural network classifier that is imposing regularization on its weights to encourage the establishment of connections that mirror the GO directed acyclic graph (DAG), and has been used to combine cancer classification with biological explanations (Bourgeais *et al.* 2021). Its successor GraphGONet directly implements the GO structure without regularization (Bourgeais *et al.* 2022). However, to our knowledge, no attempts have yet been made to incorporate full hierarchical biological networks into a VAE in order to capture the different levels of description of biological processes in tasks that go beyond classification.

Here, we introduce OntoVAE (Ontology guided VAE), a novel flexible VAE architecture with a multi-layer, sparse decoder that allows for the incorporation of any kind of hierarchical biological information encoded as an ontology. OntoVAE provides direct interpretability in its latent space and decoder, as the activities of the neurons now correspond to activities of biological processes or phenotypes. Its efficient implementation allows users to consider thousands of terms and monitor their activity changes, without the need to preselect-specific processes. Importantly, OntoVAE can be used for predictive modeling. By modulating the values of input features *in silico* and then monitoring how these changes propagate through the network, OntoVAE can simulate the effects of drug treatment or genetic alterations. The investigation of subsequent alterations in the activation of hidden nodes representing processes or phenotypes allows to uncover complex genotype–phenotype relationships. Thus, we consider OntoVAE a powerful tool for *in silico* screening approaches.

## 2 Materials and methods

### 2.1 Variational autoencoder

The VAE is a probabilistic DL model consisting of two coupled, but independently parameterized neural networks: an encoder, which learns a probability distribution over a compressed representation of the high-dimensional input data, which we refer to as latent space, and a decoder, which tries to reconstruct the original input from this latent space representation after sampling from the learnt distribution (Kingma and Welling 2013). More formally, the encoder part, or recognition model, yields a posterior distribution $q_{\phi }(z/x)$, with θ being the learnable parameters of a neural network, *x* the input data and *z* the latent space. The decoder part, or generative model, yields a likelihood distribution $p_{\phi }(x/z)$, with ϕ being the learnable parameters of a neural network (Kingma and Welling 2019). During the training process, both parts are jointly optimized using stochastic gradient descent. As computation of the posterior is intractable, the optimization objective of the VAE, like of other variational inference methods, is the evidence lower bound (Kingma and Welling 2019, Seninge *et al.* 2021):


(1) $$L_{\phi ,\theta }(X)=E_{q_{\phi }(z/x)}[\log p_{\phi }(x/z)]-D_{KL}(q_{\phi }(z/x)||p_{\theta }(z)).$$

The first part of Equation (1) is trying to optimize the likelihood of the observed data under a given model. The second part of Equation (1) is the Kullback–Leibler (KL) divergence, which determines the divergence of the approximate posterior from the true posterior. In practice, the true posterior $p_{\theta }(z)$ is often modeled as a multivariate normal distribution, thus, the KL divergence has a closed-form solution (Kingma and Welling 2013). The reparameterization trick is applied during model training to enable standard backpropagation (Kingma and Welling 2013).

### 2.2 OntoVAE architecture

**Figure 1.**
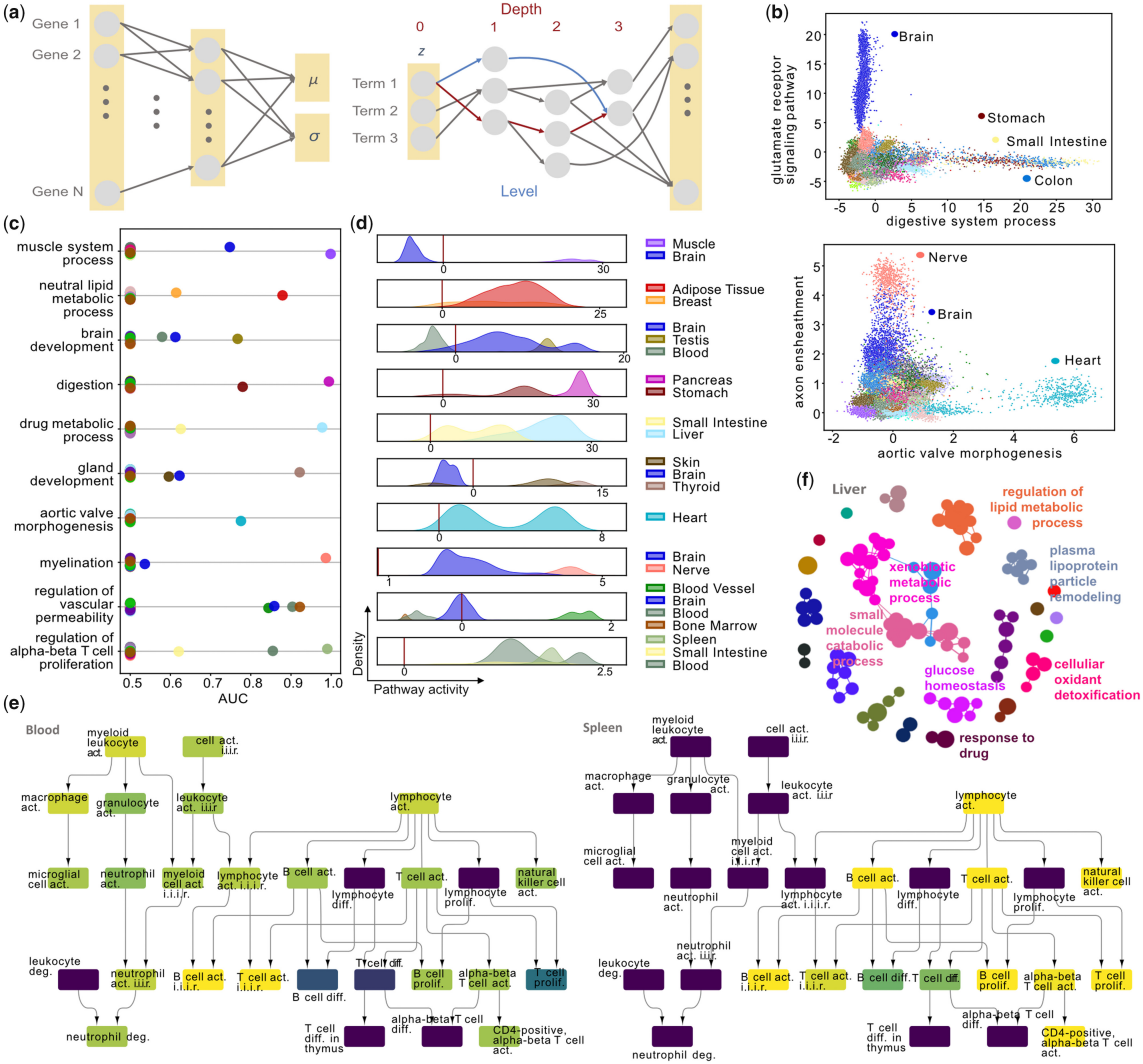
OntoVAE—a novel VAE architecture that can incorporate biological ontologies and provide pathway activities in its latent space and decoder. (a) Schematic overview of OntoVAE. A non-linear encoder is coupled to a masked, multi-layer linear decoder reflecting a biological ontology. The latent space incorporates the root terms of the ontology, each layer in the decoder represents one depth of the ontology, with “depth” referring to the longest possible path between node and a root node and “level” referring to the shortest possible path between a node and a root node. (b–e) OntoVAE with GO-decoder has been trained on all tissue samples from GTEx. (b) Scatter plots showing example pathway activations for digestive system process and glutamate receptor signaling pathway (top), and aortic valve morphogenesis and axon ensheathment (bottom). (c and d) Naive Bayes classifier with 10-fold cross-validation was trained for each GO term for each tissue (1-vs-all). Median AUC for each tissue for 10 example pathways is displayed in (c), for tissues with AUC higher than 0.5, density over the pathway activity is displayed in (d), with the vertical line corresponding to a pathway activity of zero. (e) Subnetwork of the GO graph, nodes are colored by the median AUC for blood (left) and spleen (right). act., activation; diff., differentiation; prolif., proliferation; deg., degranulation; i.i.i.r., involved in immune response. (f) Top GO term network for liver. Top GO terms were identified using pairwise Wilcoxon testing at each term and then further summarized into networks based on their semantic similarities.

OntoVAE is a modified VAE whose latent space and decoder are implemented so that they can incorporate any DAG, such as a biological ontology (Fig. 1a). For this manuscript, we used GO and Human Phenotype Ontology (HPO). A DAG is suitable for incorporation into a DL model due to its hierarchical structure and some of its main properties, such as the absence of connected loops and the possibility of a child term to have multiple parent terms (unlike a tree). Note the definition of “level” and “depth” here, with “level” referring to the shortest possible path between a node and a root node, and “depth” referring to the longest possible path between a node and a root node. For ontology incorporation into the model, we define the latent space as the root layer (depth 0), and each layer of the decoder corresponds to one depth level of the ontology, meaning that terms of the ontology that have the same depth will be located in the same layer. Thus, the most generic terms are located in the latent space, and terms become more and more specific with progression through the decoder until reaching the reconstruction layer, which is made up of genes. Note that, although we only used gene expression data in this project, in principle, any type of data can be used as input and output that can be mapped to the terms of the ontology used, such as DNA methylation or SNP data. A DAG usually has one root node, but because a 1D latent space would not be meaningful, we apply a trimming process to our ontologies before incorporation, where the most generic terms, with too many annotated genes, as well as the most specific terms, with too few annotated genes, are removed (Supplementary Fig. S1). It should be noted that trimming leads to a less deep model and can change the depth annotation of a term, and therefore the ontology layer that a term is located in. Thresholds for pruning can be chosen by the user.

The decoder of OntoVAE is linear, multi-layered and sparse, as it only models connections between parent and child terms of the ontology as well as between terms and annotated genes. In contrast to a standard VAE where connections are only present between neighboring layers, OntoVAE also has to model connections between non-neighboring layers as well as between any layer and the reconstruction layer, as terms in each depth level have genes annotated to them. This is achieved by a successive layer concatenation process similar to the skip connections in DenseNet (Huang *et al.* 2016) when passing samples through the model, where at each step, the values from the previous decoder layer are concatenated to the current one (see Supplementary Fig. S2 for a schematic drawing). Furthermore, at each step, a binary mask is used to set the weights of non-existent connections to zero. Thus, in principle, connections can be encoded between any two pairs of layers of a given ontology.

We postulate that the activation value, we retrieve from a neuron located in the latent space or decoder of the model when running samples through the trained model corresponds to the pathway activity of the term represented by this neuron. To preserve directionality in the pathway activities, we restrict the weights of our decoder to be positive. Furthermore, we use three neurons per term to compensate for the orientation of the ontology in the model, with information flowing from parent to children terms, and reduce correlations between siblings that share the same parent nodes and would therefore receive the same input if only one neuron per term was used (Supplementary Fig. S4e, left panel). This approach also helps in reducing the correlations between terms with high Jaccard similarity (js) (Supplementary Fig. S4e, right panel). Thus, in order to obtain the pathway activity for one term, we take the average of the activities of the three neurons representing the term.

### 2.3 Ontologies and datasets

The preprocessed ontologies and datasets used in the manuscript are provided under https://figshare.com/projects/OntoVAE_Ontology_guided_VAE_manuscript/146727. Ontologies were downloaded in obo format and parsed with the function get_godag() from the Python package goatools (v 1.0.15).

#### 2.3.1 Gene Ontology

Ontology was downloaded from geneontology.org (go-basic.obo, release date: 1 February 2021) together with the GAF annotation file (goa-human.gaf, version 2.1) (Ashburner *et al.* 2000). We filtered everything for the “Biological Process” sub-ontology and kept only “is a”-relationships between terms. We furthermore trimmed the ontology using a bottom threshold of 30 and a top threshold of 1000 (Supplementary Fig. S1), ending up with 3083 GO terms and 19 469 annotated genes when using HGNC symbols, and 3245 GO terms and 19 387 annotated genes when using Ensembl IDs. HGNC symbol to GO term annotations were extracted from the GAF file. To obtain Ensembl ID to GO term annotations, Uniprot IDs from the GAF file were converted to Ensembl using a mapping file from ftp.uniprot.org (idmapping_selected.tab, download: April 2021).

#### 2.3.2 Human Phenotype Ontology

The ontology was downloaded from hpo.jax.org (hp.obo, release date: 14 April 2022) together with an annotation file (genes_to_phenotype.txt) containing the mapping of HPO IDs to genes (Robinson *et al.* 2008). We applied the same preprocessing and trimming steps as for GO, but this time using thresholds of 10 and 1000, and ended up with 4525 HPO terms and 4774 annotated genes represented by HGNC symbols.

#### 2.3.3 Genotype Tissue Expression dataset

Genotype Tissue Expression (GTEx) bulk RNA-seq data from human tissues was retrieved through the R package recount3 (v 1.1.8), which is communicating with recount3, a database of uniformly reprocessed RNA-seq datasets (Carithers *et al.* 2015, Wilks *et al.* 2021).

#### 2.3.4 Limb-girdle muscular dystrophy dataset

Bulk RNA-seq data sequenced from muscle samples of limb-girdle muscular dystrophy (LGMD) patients and healthy individuals were downloaded from Gene Expression Omnibus (accession number: GSE202745) (Depuydt *et al.* 2022).

#### 2.3.5 Peripheral blood mononuclear cells dataset

The preprocessed single-cell RNA-seq dataset of interferon (IFN)-ß treated peripheral blood mononuclear cells (PBMCs) was downloaded through the github repository of VEGA (https://github.com/LucasESBS/vega-reproducibility) (Kang *et al.* 2018).

### 2.4 Model implementation and training

The OntoVAE framework is written in pytorch. All OntoVAE models in this manuscript have been trained with a batch size of 128, a dropout of 0.2 in the hidden layer of the encoder, a dropout of 0.5 on the latent space layer, and a weighting coefficient of 1e-4 on the KL loss. Model training was performed on 80% of the samples, while the remaining 20% were used for validation. Models were usually trained over 300 epochs, and the model with the best validation loss was used. AdamW was used as the optimizer together with a learning rate of 1e-4. The dimensions of latent space and decoder are always defined by the used ontology. Three neurons were used per ontology term. The following pretrained models are provided under https://figshare.com/projects/OntoVAE_Ontology_guided_VAE_manuscript/146727: (i) GO with Ensembl IDs trained on GTEx bulk RNA-seq data downloaded through recount3, (ii) HPO with Gene Symbols trained on GTEx bulk RNA-seq data downloaded through recount3, and (iii) GO with Gene Symbols trained on unstimulated cells from the PBMC dataset. The exact layer compositions of the three models are given in Supplementary Tables S1–S3.

### 2.5 Retrieval and comparison of pathway activities

We retrieve the activities at each node when running samples through a pretrained model by attaching pytorch hooks to each layer. To compare pathway activities between groups of samples, we performed two-tailed Wilcoxon tests using the stats.ranksums function from the scipy package (v 1.5.2) with a *P*-value cutoff of .05 at each node in the decoder.

### 2.6 Classification of GTEx tissues based on pathway activities

For each GO term and each tissue, we trained a 1-vs-all naive Bayes classifier with 10-fold cross-validation and computed the median area-under-the-curve (AUC) from the ten folds. Subnetworks of the GO graph that are represented in Fig. 1e and colored by the median AUC were generated with CytoScape (v1.9.3).

### 2.7 Creation of GO networks

For the creation of tissue-specific GO networks for the GTEx dataset (Fig. 1f), we performed Wilcoxon tests at each node/for each term for all possible two pairs of tissues, and called it a hit if a term was significantly more active in one tissue compared to another (*P*-value ⩽ .05). We then counted the number of hits per tissue and used this as a measure to rank all tissues for one term, with the tissue with most hits receiving Rank 1 for this term, the tissue with second most hits receiving Rank 2 and so on. For a given tissue, the terms were then sorted again based on their rank, the number of hits, and the median of the Wilcoxon test statistic. We then took the top 100 terms of the tissue and clustered them based on their Wang semantic similarities (Wang *et al.* 2007). Thus, each node in the network corresponds to one GO term, the size of a node reflects the number of genes associated with a term. If all terms in a cluster had a common ancestor, this ancestor term was chosen as cluster representative. Otherwise, the term with most annotated genes was chosen as cluster representative. GO networks for all tissues can be explored in our Dash-based web application (http://ontovaemodelexplorer.pythonanywhere.com/). The rankings of the GO terms for all tissues can be found in the Supplementary Material.

### 2.8 Calculation of js

For all possible pairs of GO terms, we compared their sets of descendant genes. We use the term “descendant genes” to specify the set of genes that is either directly annotated to a given term or to one of its descendant terms. js was calculated by dividing the number of elements in the intersection of the two sets by the number of elements in their union. We then defined three intervals (low: js ⩽ 0.25, medium: 0.25 < js ⩽ 0.75, high: js < 0.75), and 1000 random term pairs were sampled from each class to produce the boxplots in the right panel of Supplementary Fig. S4e.

### 2.9 Simulation of genetic and drug-induced perturbations

All simulations in this study were carried out in a single-gene context, meaning that we only perturbed the expression of one gene at a time. For the study of genetic perturbations, *in silico* gene knockouts have been performed by setting the input value for a gene to zero before running data through the trained model and retrieving pathway activities. For the study of drug-induced perturbations, *in silico* stimulation of genes was performed by increasing their input value prior to running data through the trained model.

#### 2.9.1 GTEx muscle + GO

Knockout of Duchenne muscular dystrophy (DMD), COX5A, and SFSWAP was performed in the 881 muscle samples.

#### 2.9.2 GTEx muscle + HPO

Systematic knockout of all 4774 genes annotated to HPO was performed in the 881 muscle samples.

#### 2.9.3 CD4T cells (unstimulated) from PBMC dataset + GO

Systematic stimulation of all 5472 genes annotated to GO and expressed in the unstimulated cells of the PBMC dataset was performed by setting their input value to 8, as 6.8 was the maximum expression value found in the unstimulated cells.

### 2.10 Perturbation analysis (paired Wilcoxon test)

For analysis of perturbation consequences, one-tailed paired Wilcoxon tests were performed between the same set of samples pre- and post-perturbation using the function stats.wilcoxon from the scipy package (v 1.5.2).

#### 2.10.1 GTEx muscle + GO

**Figure 2.**
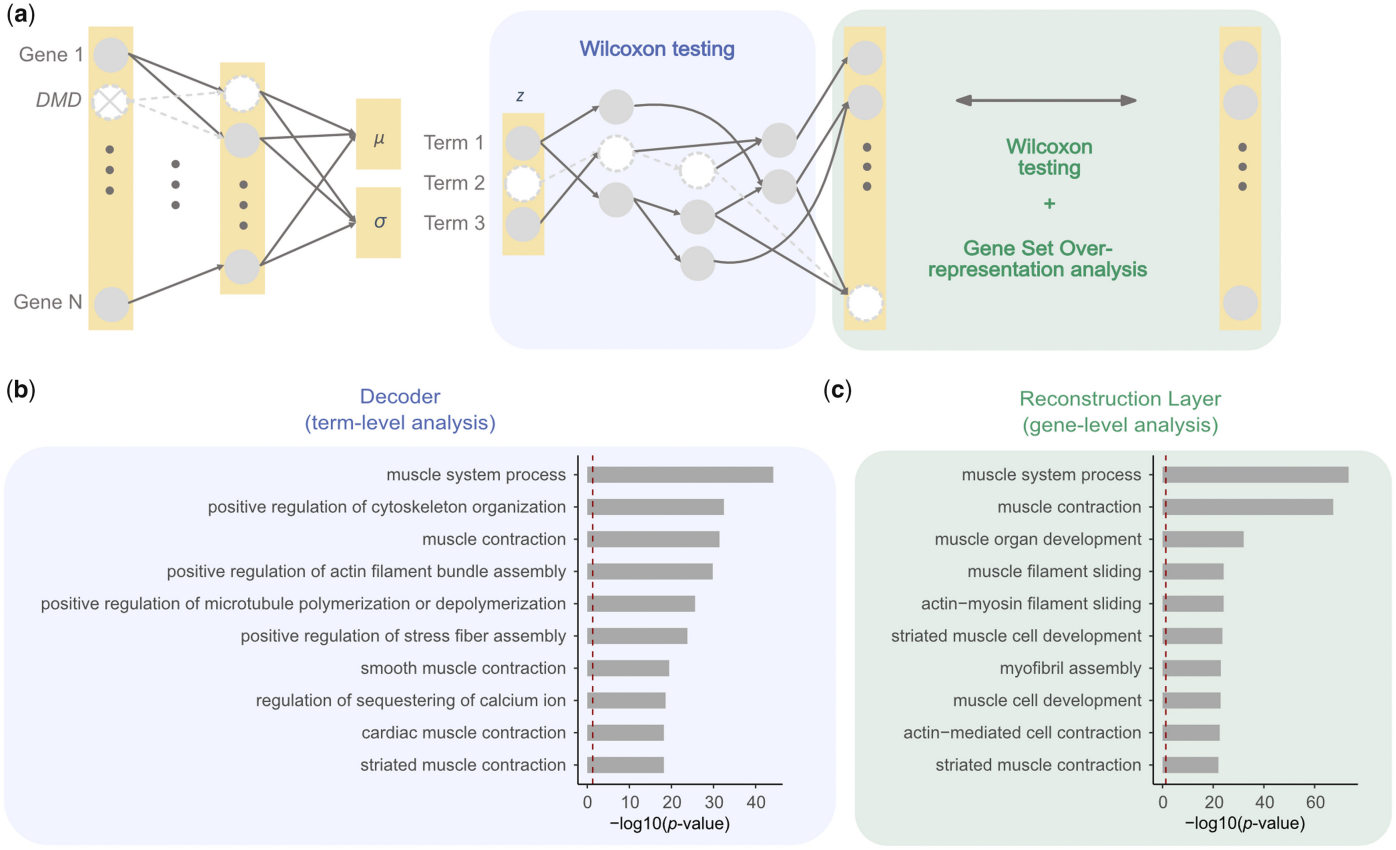
OntoVAE can predict phenotypic outcome of a gene knockout (here: DMD). OntoVAE has been trained with a GO-decoder on all GTEx tissue samples, knockouts have been performed in muscle samples only. (a) Schematic drawing of how the model can be used for *in silico* investigation of gene knockouts. Input value for a gene (here: DMD) can be set to zero before running samples through the trained model and obtaining their activations at each node/term. Paired Wilcoxon tests can be performed for all the terms in latent space and decoder (pre-knockout versus post-knockout) to identify the most affected terms (term-level analysis). Paired Wilcoxon tests can also be performed for all the genes in the reconstruction layer to identify the most affected genes, and these can then be further grouped into terms using gene set ORA (gene-level analysis). (b) Knockout of DMD in GTEx muscle samples. Barplot displays the 10 most affected terms from the term-level analysis ranked by significance. (c) Knockout of DMD in GTEx muscle samples. Barplot displays the 10 most affected terms from the gene-level analysis ranked by significance. (b and c) The dotted line represents the significance threshold.

Tests were performed at each node in latent space and decoder to identify terms significantly downregulated upon knockout (term-level analysis, Fig. 2b). Tests were also performed for each gene in the reconstruction layer to identify genes significantly downregulated upon knockout (gene-level analysis, Fig. 2c).

#### 2.10.2 GTEx muscle + HPO

Tests were performed specifically at the decoder node LGMD in both directions, to identify genes that significantly up- or downregulated the activity of this node. Genes were then ranked according to their *P*-value.

#### 2.10.3 CD4T cells (unstimulated) from PBMC dataset + GO

Tests were performed specifically at the decoder node Type I IFN signaling pathway to identify genes that significantly upregulated the activity of this node. Cells that originally had zero expression of a particular gene were not included in the test. The number of cells that was included per gene can be found in Supplementary Table S4. Genes were then ranked according to their *P*-value.

### 2.11 Differential gene expression analysis

#### 2.11.1 PBMC dataset

Differential gene expression analysis (DGEA) was performed between IFN stimulated and unstimulated cells for each cell type separately. Only genes were included that could also be mapped to GO. We performed two-tailed Wilcoxon tests using the stats.ranksums function from the scipy python package (v 1.5.2) and corrected for multiple testing with the function stats.multitest.fdrcorrection from the python package statsmodels (v 0.12.0). For each cell type, we then selected genes significantly upregulated upon IFN treatment, using an FDR threshold of 0.1. Gene set upregulated in CD4T cells was termed CD4T_IFN-ß_stim_up.

#### 2.11.2 LGMD dataset

DGEA was performed between muscle samples of patients (*n* = 42) and controls (*n* = 33) using the R package DESeq2 (v 1.26.0). Eight patient samples and one control sample had been excluded as outliers prior to analysis after PCA inspection of the dataset. We used a significance threshold of 0.1 for the adjusted *P*-value and kept only genes that could be mapped to HPO. Genes upregulated in LGMD muscles compared to control were termed LGMD_up, genes downregulated in LGMD muscles compared to control were termed LGMD_dn.

### 2.12 Gene set enrichment analysis

Gene set enrichment analysis (GSEA) was performed with the R package clusterProfiler (v 3.14.3).

### 2.13 Gene set overrepresentation analysis

Overrepresentation analysis (ORA) was performed with the R package clusterProfiler (v 3.14.3).

### 2.14 Cell clustering and adjusted rand index computation

To compare gene expression, OntoVAE + GO, OntoVAE + Reactome, VEGA + Reactome, and expiMap + Reactome, cells were clustered using Leiden clustering (scanpy v1.9.3) with the resolution hyperparameter set to 0.2, 0.2, 0.4, 0.3, and 1.2, respectively. Adjusted rand index (ARI) was then computed with the adjusted_rand_score function from scikit-learn (v1.0.2).

## 3 Results

### 3.1 Architecture of OntoVAE

To make the latent space and decoder part of a VAE model biologically interpretable, we created a novel architecture that we named OntoVAE where we implemented the latent space and decoder in a way that any biological ontology, such as GO or HPO, can be incorporated (Fig. 1a). Thus, every node in the latent space and every node in the decoder represents a term of the ontology, with root terms being located in the latent space and terms becoming more and more specific with progression through the decoder. Originally, every ontology has a common root node, but as a 1D latent space would not be meaningful, we apply a trimming process to the ontology, where we remove the root and other very generic terms that have a high number of annotated genes. The resulting zero depth terms after this trimming define the latent space. We furthermore remove very specific terms with too few annotated genes. Details on this pruning process can be found in Section 2 and Supplementary Fig. S1.

Following the lines of previous research (Svensson *et al.* 2020, Seninge *et al.* 2021), the decoder of our model is linear, meaning that no activation functions are used in the layers, which trades off reconstruction accuracy for interpretability. The decoder is also sparse, as connections are only modeled between parent and children ontology terms as well as between terms and annotated genes. To be able to represent connections between non-neighboring layers, we implemented skip connections through concatenation in our model, following the principle introduced in DenseNet (Huang *et al.* 2016). Our approach is illustrated in Supplementary Fig. S2 (also see Section 2). Furthermore, we restrict all the weights in the decoder to be positive to preserve directionality in the model even when trained several times as has already been done in VEGA (Seninge *et al.* 2021). To model more complex relationships, each term of the ontology is modeled by three neurons.

### 3.2 OntoVAE provides biological interpretability in latent space and decoder

We postulate that the activations of the neurons in the latent space and decoder can be directly interpreted as pathway or phenotype activities of the corresponding ontology. To demonstrate that, we incorporated the GO into our model and trained it on the GTEx bulk RNA-seq data (Ashburner *et al.* 2000, Harris *et al.* 2004, Carithers *et al.* 2015). We then retrieved the activities of all terms in the latent space and decoder for each sample and looked at some example terms to see if they were more active in the expected tissues. We found that, for instance, digestive system process is especially active in stomach, colon, and small intestine, glutamate receptor signaling pathway is especially active in brain, axon ensheathment is especially active in nerve, and aortic valve morphogenesis is especially active in heart (Fig. 1b).

Next, we wanted to investigate in a more systematic manner whether the obtained pathway activities could be used to classify the correct tissues. For this purpose, we trained a naive Bayes classifier with 10-fold cross-validation for each GO term and each tissue in a 1-vs-all setting. We then calculated the median AUC, which is summarized for all tissues and pathways in the Supplementary Material and displayed for 10 example pathways in Fig. 1c. As expected, for majority of the tissues, the AUC is 0.5, which corresponds to random classification, and a higher AUC is achieved for the correct tissues, e.g. for adipose tissue and breast in neutral lipid metabolic process, for pancreas and stomach in digestion, or for liver and small intestine in drug metabolic process. However, for some pathways, we find unexpected tissues, e.g. brain for muscle system process and gland development, or blood for brain development. When we looked at the density of these tissues over the pathway activities, we observed that the high AUC in these cases is due to a very low pathway activity of this tissue (Fig. 1d). A heatmap displaying the top 10 GO terms with AUC higher than 0.5 is illustrated in Supplementary Fig. S3.

To exploit the hierarchy that is incorporated in our model by design, we also set out to compare how different samples can follow different trajectories through the decoder graph. We mapped the median AUCs back to the ontology, and display an example of a trimmed subnetwork of the GO graph in Fig. 1e, where the nodes are colored either by the median AUCs for blood tissue (left graph) or for spleen tissue (right graph). The subnetwork can be divided into two main branches, one consisting of terms related to the myeloid lineage, and the other consisting of terms related to the lymphoid lineage. While both branches can classify blood tissue, spleen is only classified accurately by the lymphoid branch. This also highlights that different depths of the ontology provide different information. For example, the node B cell activation involved in immune response shows a high AUC in both tissues, while parent terms have very divergent AUCs.

Because a high AUC does not necessarily correspond to a high pathway activity, we took a different approach in extracting the top GO terms for a given tissue. We performed Wilcoxon tests between all possible pairings of tissues at each node, and then assigned a rank to each term for each tissue (see Section 2), allowing us to further group the terms that were most relevant for a given tissue into a network based on their Wang semantic similarities. All results can be found in the Supplementary Material and as an example, we show the network for liver tissue (Fig. 1f), where we find clusters related to regulation of lipid metabolic process, response to drug, xenobiotic metabolic process, and glucose homeostasis. This again confirms that our model generates meaningful biological results. The pathway activities for all GO terms as well as the networks for all tissues can be interactively explored in our web application (http://ontovaemodelexplorer.pythonanywhere.com).

### 3.3 OntoVAE generates reproducible results

In order for the previous results to be biologically meaningful, we should obtain similar results every time we train the same model with the same hyperparameters. To confirm this, we trained the model 10 times using the same hyperparameters, and using either one, two or three neurons per term, resulting in 30 trained models. For each GO term, we then calculated the Pearson correlation with itself when retrieving its pathway activities from two different models and found that the majority of correlations was higher than 0.95, thus ensuring the reproducibility of OntoVAE (Supplementary Fig. S4a).

In the context of these investigations, we also wanted to explore how the model would perform if the ontology information was integrated in the encoder. Thus, we performed the same experiment with a GO-encoder model and found the results to be less reproducible (Supplementary Fig. S4a) and the agreement between the GO-encoder and GO-decoder model to be worse (Supplementary Fig. S4b), which led us to use an ontology inspired decoder for the remaining manuscript.

We additionally investigated the influence of using different trimming thresholds with the same approach of correlating pathway activities between two models, and found that thresholds of 1000/30 (top/bottom) and 1200/10 showed a good agreement, while 800/50 showed worse correlations with the other thresholds (Supplementary Fig. S4c). We hypothesized that due to the stringent trimming, too much information from the ontology was lost. So, our chosen default threshold 1000/30 represents a good compromise in reducing complexity (Supplementary Fig. S4d) and preserving true biological signals. The trimming procedure leads to a less deep model; however, we slightly increase model complexity again by using three neurons per term in latent space and decoder. Modeling each term with more than one neuron reduces the number of highly correlated pairs of terms (Supplementary Fig. S4e, left panel, also see Section 2). We also computed the js between all pairs of terms and show that more similar terms are also more correlated in terms of their activity, although this effect is somewhat mitigated by increasing the number of neurons per term (Supplementary Fig. S4e, right panel, also see Section 2).

We also investigated the validation loss of our model and how it changes in dependence of the number of neurons per term (Supplementary Fig. S4f) and the chosen trimming thresholds (Supplementary Fig. S4g) compared to the standard VAE. As expected, the loss is worse for OntoVAE, since we are trading off accuracy for interpretability, but can be improved by using more neurons per term, since this is increasing the number of learnable parameters.

### 3.4 OntoVAE can be used for *in silico* phenotype predictions of gene knockouts

Next, we investigated whether OntoVAE could be used for predictive modeling and simulate the outcome of a gene knockout. For this purpose, we performed an *in silico* knockout of the DMD gene in the GTEx muscle samples. The DMD gene encodes for the protein dystrophin, which is located primarily in muscles and attaches the cytoskeleton to the extracellular matrix. Depletion of functional dystrophin protein due to a mutation in the DMD gene causes DMD, a genetic disease leading to muscle weakness and muscle degradation (Nowak and Davies 2004).

The model was trained on all samples from all GTEx tissues. Then, the knockout was performed by setting the input value for the DMD gene to zero before applying the trained model on the muscle samples and obtaining the activity values (Magnusson *et al.* 2022). We then used paired Wilcoxon tests at each node in latent space and decoder to compare which term activities significantly differed post-knockout (term-level analysis). We also computed paired Wilcoxon tests for each gene in the reconstruction layer to identify the most significantly affected genes, and performed ORA on the top 100 genes to further group them into GO terms (gene-level analysis) (Fig. 2a). From the term-level analysis, we obtained terms that are highly related to DMD function, such as muscle system process, positive regulation of cytoskeleton organization, and muscle contraction (Fig. 2b). The gene-level analysis also yielded DMD-related terms, such as muscle system process, muscle contraction, and muscle organ development (Fig. 2c). These results confirm our hypothesis that OntoVAE models meaningful relationships between the genes and can thus be used to predict the consequences of a gene knockout, with direct interpretability on a pathway level in latent space and decoder. We also wanted to show that these muscle-related terms are not the result of the fact that we used only GTEx muscle samples. Hence, as a negative control, we also performed the knockout for SFSWAP, a splicing factor, and COX5A, an enzyme of the mitochondrial respiratory chain, and obtained terms highly related to the respective gene (Supplementary Fig. S5). All results of the paired Wilcoxon tests and the ORA can be found in the Supplementary Material.

### 3.5 OntoVAE can predict disease-specific gene expression changes

**Figure 3.**
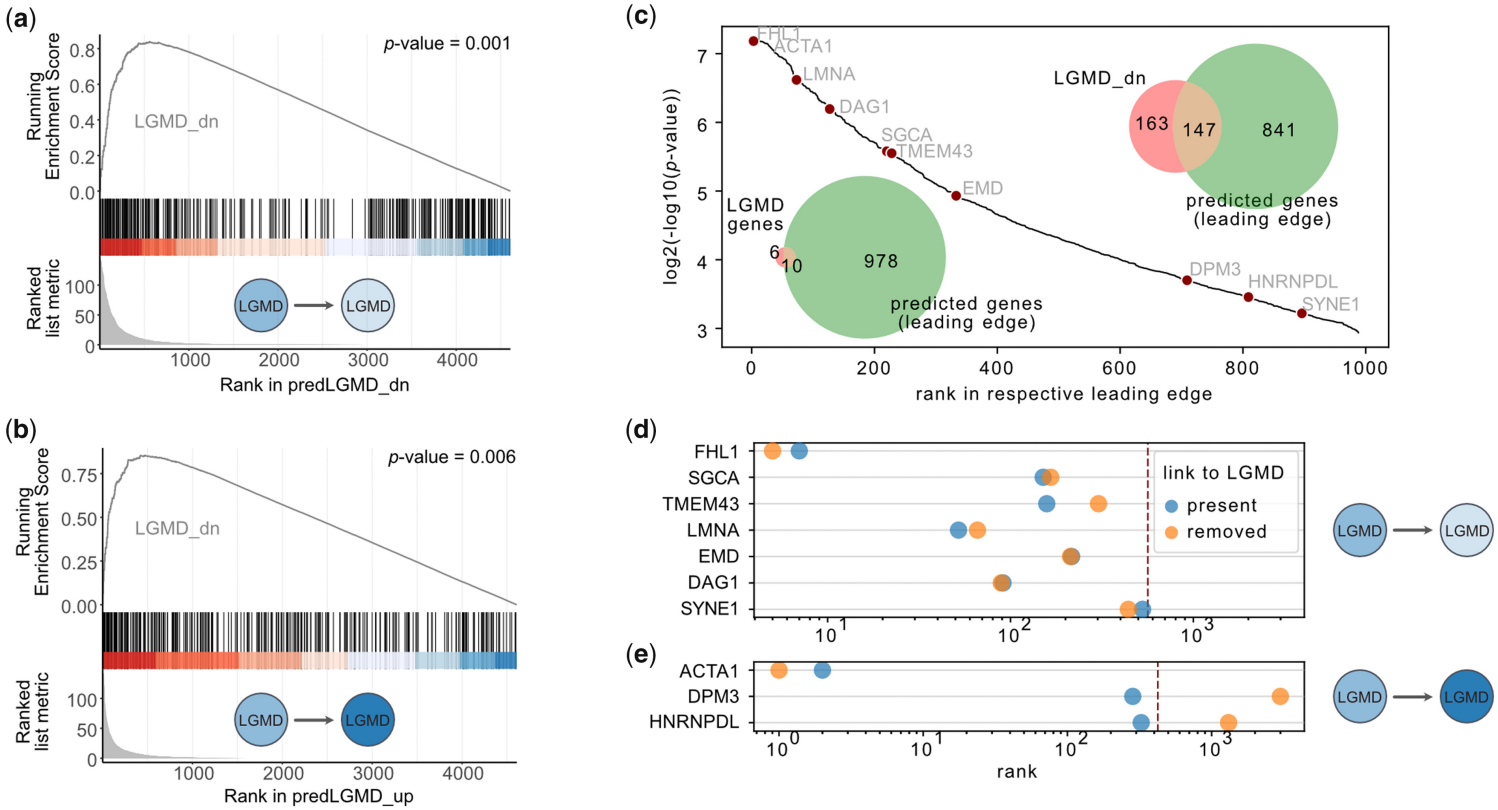
OntoVAE can predict differential gene expression in disease. OntoVAE has been trained with HPO-decoder on all tissue samples from GTEx, knockouts were performed in muscle samples only. Knockout was performed for all genes one-by-one by setting their input value to zero, and one-tailed paired Wilcoxon tests at the LGMD node in the decoder were computed for each gene, comparing pre- and post-knockout. All genes were then ranked according to the *P*-value of the paired Wilcoxon test, and GSEA was performed with the genes that were upregulated in patients versus control (LGMD_up) and the genes that were downregulated in patients versus control (LGMD_dn) in the LGMD dataset. (a) GSEA results using ranking of genes that significantly downregulated the LGMD node in the decoder. (b) GSEA results using ranking of genes that significantly upregulated the LGMD node in the decoder. (c) Leading edges of both GSEAs were fused (predicted genes). Venn diagram in the top right corner shows overlap between the predicted genes and LGMD_dn. Venn diagram in the bottom left corner shows overlap between predicted genes and genes directly annotated to the term LGMD in HPO. Hockeystick plot displays the ranking of the predicted genes, the 10 genes directly annotated to LGMD in HPO are labeled. (d and e) For each of the 10 genes, a new model was trained where the link of the gene to LGMD had been removed. Plots show the ranking of the 10 genes before (blue circle) and after (orange circle) removing the link to LGMD. (d) Genes that were found in the leading edge of (a), dotted line indicates leading edge cutoff. € Genes that were found in the leading edge of (b), red dotted line indicates leading edge cutoff.

Next, we asked whether OntoVAE could actually predict differential gene expression in the context of disease. As a use-case, we selected a form of muscular dystrophy, LGMD, in order to evaluate to what extent our model can predict differentially expressed genes between disease and healthy control samples. We adapted our model to use the HPO (Robinson *et al.* 2008), which consists of disease-related terms, among them the term LGMD, a child node of the more generic term muscular dystrophy. To predict the genes that most significantly influence the LGMD node in the decoder, we systematically performed a knockout for all genes one-by-one in the GTEx muscle samples by setting their input value to zero before passing the samples through the trained model, and then computed a paired Wilcoxon test at the LGMD node for each gene, comparing the activities at this node before and after knockout of the gene. For HPO, the sign of the influence of a gene on a term (either enhancing or inhibiting the phenotype) is not clearly defined. Therefore, we looked at both directionalities, and performed two separate one-tailed paired Wilcoxon tests for each gene, and then ranked the genes according to their *P*-values, ending up with two ranked lists: one list ranking the genes that downregulated the LGMD node upon knockout (predLGMD_dn) (Fig. 3a and Supplementary Material), the other list ranking the genes that upregulated the LGMD node upon knockout (predLGMD_up) (Fig. 3b and Supplementary Material).

To verify the validity of these predictions, we performed a GSEA using as a ground truth the differentially expressed genes in a recently published dataset of bulk RNA-seq carried out on muscle samples from LGMD patients (*n* = 16) and healthy individuals (*n* = 15) (Depuydt *et al.* 2022), where we had determined the genes that were significantly up- (LGMD_up) or downregulated (LGMD_dn) in patients compared to age-matched controls (Supplementary Material). For both lists (predLGMD_up and predLGMD_dn), we found a significant enrichment of LGMD_dn (Fig. 3a and b), confirming that OntoVAE is able to predict differentially expressed genes between disease and control. We fused the genes from the leading edges of the two GSEA analyses, and checked the overlap of those predicted genes with LGMD_dn, and with genes directly annotated to the term LGMD in the HPO (Fig. 3c). We found an overlap of 147 and 10, respectively. Next, we were interested to verify if the 10 genes directly annotated to LGMD ended up in the leading edge of the prediction as a result of their direct annotation to LGMD. Hence, for these 10 genes, we trained 10 new models, in which we removed the link to LGMD for one of the 10 genes. We then performed the knockout again to see if the genes would still end up in their respective leading edge. Of those 10 genes, 7 had significantly downregulated the activity at the LGMD node, and were still located in the leading edge of predLGMD_dn after their link to LGMD had been removed (Fig. 3d). The remaining three genes had significantly upregulated the activity at the LGMD node, and one of them was still in the leading edge of predLGMD_up after link removal (Fig. 3e). This analysis confirms that OntoVAE can tolerate missing prior information to a certain degree, as for the majority of the genes it recovered their importance in absence of direct annotation. It also confirms that OntoVAE learns complex relationships that go beyond the direct annotation of a gene to a specific term.

### 3.6 OntoVAE can predict treatment effects *in silico*

**Figure 4.**
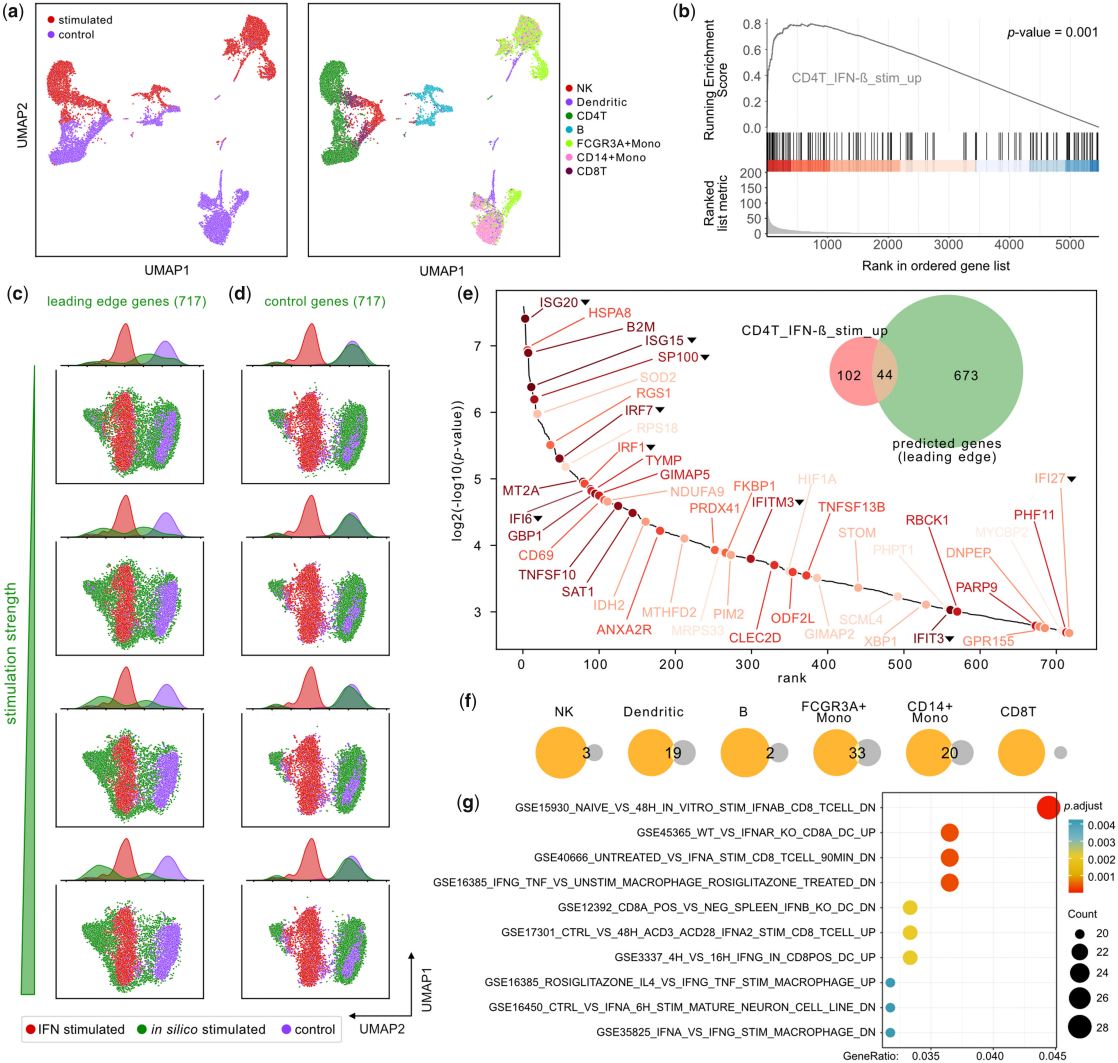
OntoVAE can predict IFN treatment response. (a) UMAP plot shows the PBMC dataset colored by cell type (right panel) and treatment (left panel). (b–e) Prediction of IFN treatment response by *in silico* stimulation of genes. All *in silico* stimulated genes were ranked according to the *P*-value of the paired Wilcoxon test, and GSEA (b) was performed with the genes that were upregulated in IFN-ß treated CD4T cells versus control CD4T cells in the original dataset (CD4T_IFN-ß_stim_up). Based on GSEA, genes from the leading edge (termed “predicted genes”) were selected. (c and d) Projection of *in silico* stimulated cells (green) on the UMAP computed using GO pathway activities for control CD4T cells (purple) and IFN-stimulated CD4T cells (red). UMAP and density plots are shown for the 717 leading edge genes (c) and the 717 bottom GSEA genes used as control (d), with stimulation strength increasing from top to bottom. Venn diagram € displaying the overlap between the predicted genes (green circle) and CD4T_IFN-ß_stim_up (red circle). € Hockey stick plot displaying the predicted genes sorted by significance, the 44 genes overlapping with CD4T_IFN-ß_stim_up are labeled. Darker label color indicates higher significance of this gene in CD4T_IFN-ß_stim_up, a black triangle next to the label indicates that the gene is directly annotated to the term type I IFN signaling pathway or to one of its descendant terms. (f and g) Further investigation of predicted genes that do not overlap with CD4T_IFN-ß_stim_up [green circle in Venn diagram € without intersection]. Venn diagrams are showing the overlap of these genes (orange circles) with the genes that are upregulated upon IFN-ß treatment in the other cell types of the PBMC dataset (gray circles) (f). Dotplot is showing results from a gene set ORA using IFN-related terms from MsigDBs C7 immunologic signature gene sets (g).

Finally, we wanted to investigate the predictive power of OntoVAE in the context of drug treatment, so we shifted back to GO to focus on IFN response. For this purpose, we used the PBMC dataset from Kang *et al.*, in which PBMCs from lupus patients were treated with IFN-ß (a type I IFN) and single-cell RNA-seq was performed (Kang *et al.* 2018). A UMAP representation of this dataset reveals clustering by treatment condition and cell type (Fig. 4a).

For our analysis, we looked at CD4T cells, as they represent the largest population from the dataset. First, we performed DGEA between stimulated and unstimulated CD4T cells and identified 146 genes that are significantly upregulated in the IFN-stimulated CD4T cells (CD4T_IFN-ß_stim_up, Supplementary Material). We used these as ground truth or “reference genes” in our analysis. We then trained our model with a GO-decoder on all unstimulated cells, and then performed one-by-one *in silico* stimulation of all input genes by setting their expression value to a higher value (see Section 2). To investigate which genes had the highest influence on the node in the GO-decoder corresponding to type I IFN signaling pathway, we computed a paired Wilcoxon test between the activations at this node before and after stimulation for each gene in the CD4T cells (Supplementary Material). The genes were then ranked according to their paired Wilcoxon *P*-value, and GSEA was performed with the gene set CD4T_IFN-ß_stim_up, yielding a significant *P*-value of .001 (Fig. 4b) and therefore confirming that OntoVAE can predict IFN response-related genes.

Next, we set out to investigate the similarity of our *in silico* perturbed CD4T cells with the IFN-stimulated (ground truth) CD4T cells. For this purpose, we retrieved OntoVAE pathway activities for CD4T control, *in silico* perturbed CD4T, and IFN-stimulated CD4T cells, computed a UMAP over the control and ground truth cells, and then projected the *in silico* cells onto that UMAP space. With increasing stimulation of the 717 leading edge genes, the *in silico* perturbed cells are approaching the ground truth cells in the UMAP space (Fig. 4c), while they remain clustered around the control cells, when the 717 bottom genes have been stimulated (Fig. 4d). Furthermore, we show that stimulation of the top 5 genes is enough to induce a shifting of cells (Supplementary Fig. S6a), and this shift is enhanced when using 20 (Supplementary Fig. S6b), 50 (Supplementary Fig. S6c), and 100 (Supplementary Fig. S6d) top genes. However, using the same numbers of random genes does not yield the same results (Supplementary Fig. S6a–d), highlighting the importance of the order of the genes in the leading edge.

We had a closer look at these 717 leading edge genes, which we refer to as “predicted genes,” and 44 of them overlapped with our ground truth CD4T_IFN-ß_stim_up (Fig. 4e), among them some of the most significant reference genes, such as ISG15, IFI6, ISG20, and B2M. Notably, of those 44 genes, 35 genes are not directly annotated to the term type I IFN signaling pathway or any of its descendant terms (Fig. 4e), showing that the model can also recover genes that are not directly linked to the node of interest. Also, among those genes, some had very low expression or were expressed in very few of the unstimulated cells in the PBMC dataset, which comprised our training data, but could still be recovered by OntoVAE (Supplementary Fig. S7). We then further analyzed the 673 predicted genes that were not overlapping with the reference genes, and found substantial overlap with the genes that were differentially upregulated upon IFN-ß treatment in some of the other cell types of the PBMC dataset, especially FCGR3A+ monocytes, CD14+ monocytes, and dendritic cells (Fig. 4f). We also performed gene set ORA with the IFN-related gene sets from MSigDBs C7 immunologic signature gene sets (Fig. 4g), finding significant enrichment of gene sets that were upregulated in CD8T cells or a mature neuron cell line upon IFN treatment, or downregulated in dendritic cells after IFN receptor knockout (first, second, third, and ninth set in Fig. 4g). Taken together, these results confirm that OntoVAE generates meaningful results through *in silico* prediction of treatment response.

### 3.7 Comparison of OntoVAE with related methods

Finally, we set out to compare OntoVAE with previously published single-layer decoder methods VEGA and expiMap (Seninge *et al.* 2021, Lotfollahi *et al.* 2023). We used the PBMC dataset again and trained VEGA and expiMap models with Reactome pathways in their latent space, following the tutorials of the authors, https://vega-documentation.readthedocs.io/en/latest/tutorials/vega_tutorial.html (as of February 2023) for VEGA and https://scarches.readthedocs.io/en/latest/expimap_surgery_pipeline_basic.html (as of February 2023) for expiMap. For comparison, we also trained OntoVAE as a single-layer model with Reactome pathways in the latent space. We then performed Leiden clustering on the four alternative methods, and directly on the gene expression data, and computed the ARI to assess the quality of the clustering (Supplementary Fig. S8a). All methods yield similar results, with expiMap performing slightly worse, however, we speculate that this is due to their higher weighting of the KL loss. Interestingly, OntoVAE coupled to the multi-layered GO-decoder shows the highest ARI values, despite a slightly higher reconstruction error compared to the single-layer Reactome version (Supplementary Fig. S8c). We also wanted to see how the single-layer methods performed on the prediction task. For this purpose, we carried out the one-by-one *in silico* gene stimulation followed by paired Wilcoxon testing at the node Reactome_Interferon_Alpha_Beta_Signaling, and again performed the GSEA with our gene set CD4T_IFN-ß_stim_up. We find that all three methods can predict the differentially expressed genes equally well (*P*-value of .001, Supplementary Fig. S8b).

## 4 Discussion

In this work, we designed OntoVAE, a novel VAE, in whose latent space and decoder any hierarchical biological network can be incorporated that has the structure of a DAG and can be trimmed to have a reasonable number of root nodes that represent the latent space variables. We applied the model on different ontologies, GO and HPO, and on a bulk RNA-seq dataset (GTEx) as well as on a single-cell RNA-seq dataset (PBMC). Using GTEx and GO, we demonstrated that the model generates reproducible results and outputs meaningful pathway activities in latent space and decoder. Furthermore, we applied OntoVAE in two scenarios of predictive modeling: genetic alterations and drug treatments. Again using GTEx and GO, we showed that we obtain meaningful terms in the decoder and reconstruction layer when simulating gene knockouts. Using GTEx and HPO, we showed how to systematically perform an *in silico* knockout of all genes to find which genes had the strongest influence on the disease LGMD, and validated our findings in an external RNA-seq dataset of LGMD. Using PBMC and GO, we showed how the model, trained on unstimulated data only, is still able to predict IFN response by systematically performing *in silico* stimulation of all genes. One interesting and central finding is the fact that the model is capable of predicting influential genes on processes or phenotypes that go beyond the ones directly annotated to the term in question, indicating that the model is capable of learning more complex gene-term relationships in a data driven way. Previous models have tackled the question of interpretable drug–response prediction, e.g. the CPA model based on AEs coupled to discriminators, which provides interpretability in terms of covariate influence, such as species or treatment dose (Lotfollahi *et al.* 2019, Hetzel *et al.* 2022). Other models, such as Dcell (Ma *et al.* 2018) and a recently published model to predict gene expression from transcription factor activities (Magnusson *et al.* 2022), have also employed synthetic knockouts. The recently published expiMap model, based on a VAE with an interpretable latent space, can also predict drug response and learn new relationships based on prior knowledge, such as pathway structure (Lotfollahi *et al.* 2023). However, unlike other models that limit the number of biological terms under scrutiny or use a single-layer linear decoder, our model is capable of encoding thousands of terms without the need for preselection and maintaining the hierarchical information contained in the ontology. In addition, our model extends to synthetic overexpression of genes to simulate drug-induced activation. As such, it uses a similar approach as the light-up procedure implemented in DeepVAE, but at the level of the input gene expression instead of the nodes of the hidden layer (Dwivedi *et al.* 2020).

One limitation of our model is that we only model one type of relationship between terms, namely the “is a” relationship, as there is no straightforward way to model different kinds of connections. By enabling more complex and diverse relationships between nodes, our current model could incorporate knowledge-graphs as a decoder in a similar fashion as we did for ontologies as an alternative to graph-neural networks that are commonly used (Li *et al.* 2022). Additionally, as we use a hard-coded decoder, the model has no possibility of correcting the prior. However, we also demonstrate that in some cases, the model can recover missing prior information, e.g. missing gene annotations to terms, suggesting that it is capable of learning complex relationships. Nevertheless, the quality of the results will heavily depend on the quality of the used prior information and adding flexibility in the connections of the decoder along the lines proposed by Rybakov *et al.* (2020) or as implemented in DeepGONet (Bourgeais *et al.* 2021) might be an interesting option to cope with incomplete annotation. For biological ontologies, one has to keep in mind that some branches are more explored than others, some terms are more central than others, with more incoming and outgoing connections, and also the genes are annotated to different numbers of terms. To which extent these parameters influence the results remains to be investigated. Furthermore, for some ontologies, such as HPO where many annotations are based on SNPs, it is challenging to interpret the results as annotated genes can have a positive or negative effect on the term, whereas our model only allows for positive weights in the decoder in order to preserve the direction when interpreting pathway activities. With our systematic knockout approach, we were only able to predict genes that were downregulated in LGMD patients, thus, further investigation is needed here. We also want to point out that the model cannot distinguish between causal and indirect relationships.

In summary, OntoVAE can be adapted to any ontology and dataset and used to compute pathway activities and to predict disease or treatment induced changes in gene expression. Our model fully exploits the conceptual complexity of the hierarchical structure of the ontology and can highlight difference between samples at different levels of the ontology. In future work, it will be interesting to also investigate the effect of multiple perturbations at a time, and see if synergistic effects can be recovered by OntoVAE. We envision that our method will be a useful first step in identifying sets of target genes or pathways, which can then be further validated experimentally.

## Supplementary Material

btad387_Supplementary_Data

## Data Availability

All datasets used for analysis in this manuscript are publicly available and listed in Section 2. Further data generated in this manuscript, such as pretrained models, preprocessed ontologies or curated gene lists, can be found either on figshare https://figshare.com/projects/OntoVAE_Ontology_guided_VAE_manuscript/146727 or in the Supplementary Material or can be made available by the authors upon request.

## Code availability

The package for preprocessing ontologies and training OntoVAE models is available at https://github.com/hdsu-bioquant/onto-vae. Code to generate the results of the manuscript is provided under https://github.com/hdsu-bioquant/OntoVAE_manuscript. A web application to interactively explore all results related to Fig. 1 of the manuscript can be accessed under: http://ontovaemodelexplorer.pythonanywhere.com.
