# Supplementary material for: Biologically informed variational autoencoders allow predictive modeling of genetic and drug-induced perturbations
Source: Bioinformatics. 2023 Jun 16;39(6):btad387. doi: 10.1093/bioinformatics/btad387 (PMC10301695; doi:10.1093/bioinformatics/btad387)
Supplement: btad387_Supplementary_Data [file btad387_supplementary_data.zip › Supplementary_Figs_and_Tables.pdf]

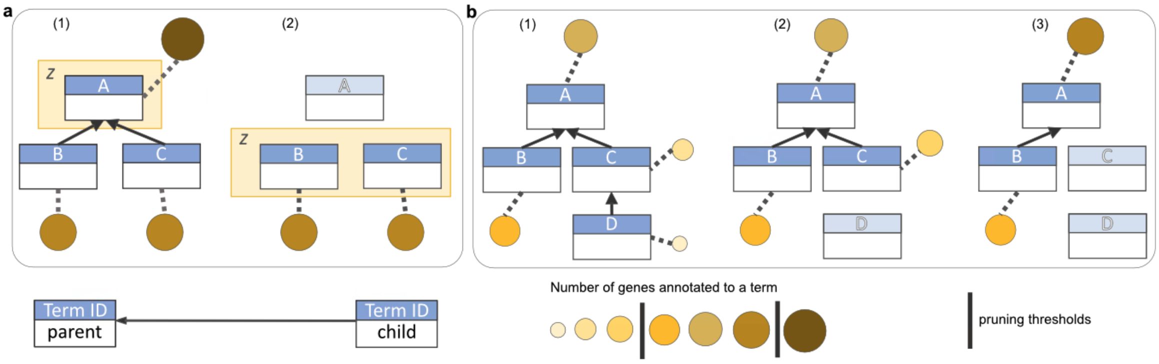

Fig. S1 Threshold-based ontology pruning for integration into OntoVAE. a Top pruning: term A is a root term located in the latent space *z* and has more genes annotated to it than the top pruning threshold (1). Thus, term A will be removed and its children terms B and C will become part of the latent space (2). **b** Bottom pruning: term D has less genes annotated to it than the bottom pruning threshold (1), so its genes are transferred to its parent term C, and D is removed (2). Term C still has less genes annotated to it than the bottom threshold, so its genes are transferred to its parent term A, and term C is pruned (3). Pruning stops here, as the number of genes annotated to A lies within the thresholds.

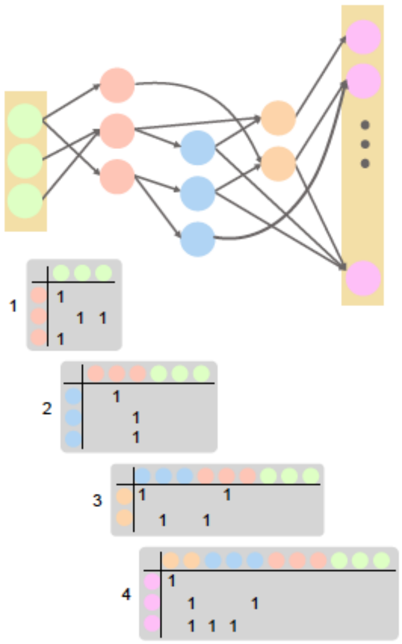

Fig. S2 Schematic drawing of skip connection modeling through concatenations. Binary masks are indicating the connections between latent space layer (green), decoder layers (red, blue, orange), and reconstruction layer (pink). At each step, the previous layer is concatenated to the current one. Mask 1 is modeling the connections between the green and the red layer, the green layer is then concatenated to the red layer, so that connections of both layers to the blue layer can be modeled by mask 2. Mask 3 is modeling the connections between the orange layer and all previous layers, mask 4 is modeling the connections between the pink layer and all previous layers.

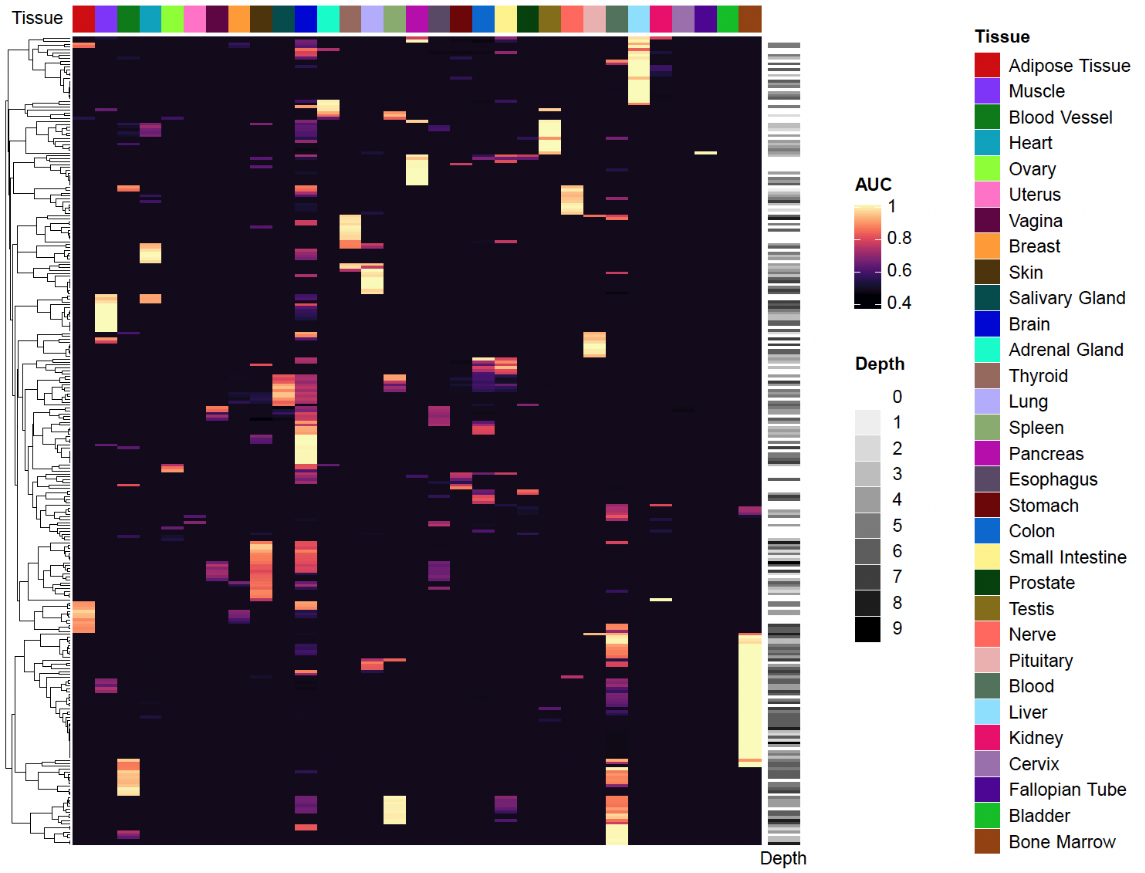

Fig. S3 Heatmap displaying top GO terms per tissue. For each GTEx tissue, the top ten GO terms with the highest median AUC were extracted given that they had a value higher than 0.5. Terms are clustered and annotated with their depth level in the trimmed ontology.

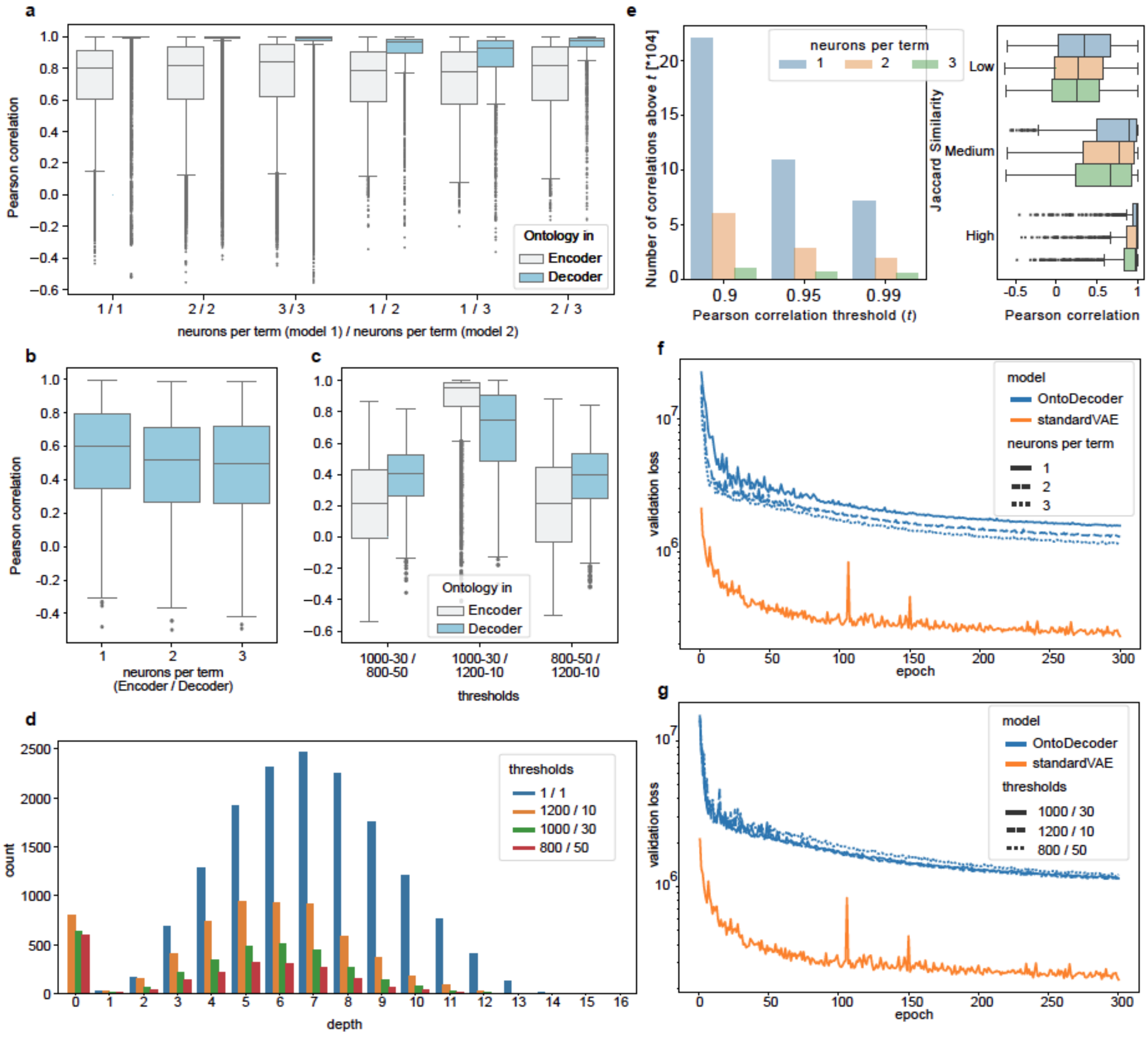

Fig. S4 OntoVAE generates reproducible results. Different models were trained with GTEx data and GO with the same hyperparameters, but using 1, 2, or 3 neurons per term, respectively, or trimming thresholds of 1000/30, 1200/10, and 800/50, respectively. **a, c** Boxplots showing the Pearson correlations for the same GO terms between different models, for models where the ontology was implemented in the encoder (light grey boxes) or on the decoder (light blue boxes). **a** For comparisons between the same number of neurons (1/1, 2/2, and 3/3), correlations were computed between 10 different models. For comparisons between different numbers of neurons (1/2, 1/3, and 2/3), correlations were computed between two different models. **b** Boxplots showing the Pearson correlations for the same GO terms between GO-encoder and GO-decoder models for different numbers of neurons per term. **c** Correlations are displayed between models with different trimming thresholds, using one trained model for each threshold. **d** Barplots showing the number of GO terms in each ontology depth before trimming (1/1) and after applying different trimming thresholds (1000/30, 1200/10, and 800/50). **e Left panel:** Barplots showing the number of pairwise Pearson correlations in the GO-decoder VAE above a certain threshold for 1, 2 and 3 neurons per term. Right panel: Boxplots showing pairwise pearson correlations for different jaccard similarities between two terms, again distinguishing between 1, 2, and 3 neurons per term. **f, g** Validation loss curves comparing the OntoDecoder model to the standard VAE for different numbers of neurons per term (**f**) and different trimming thresholds (**g**).

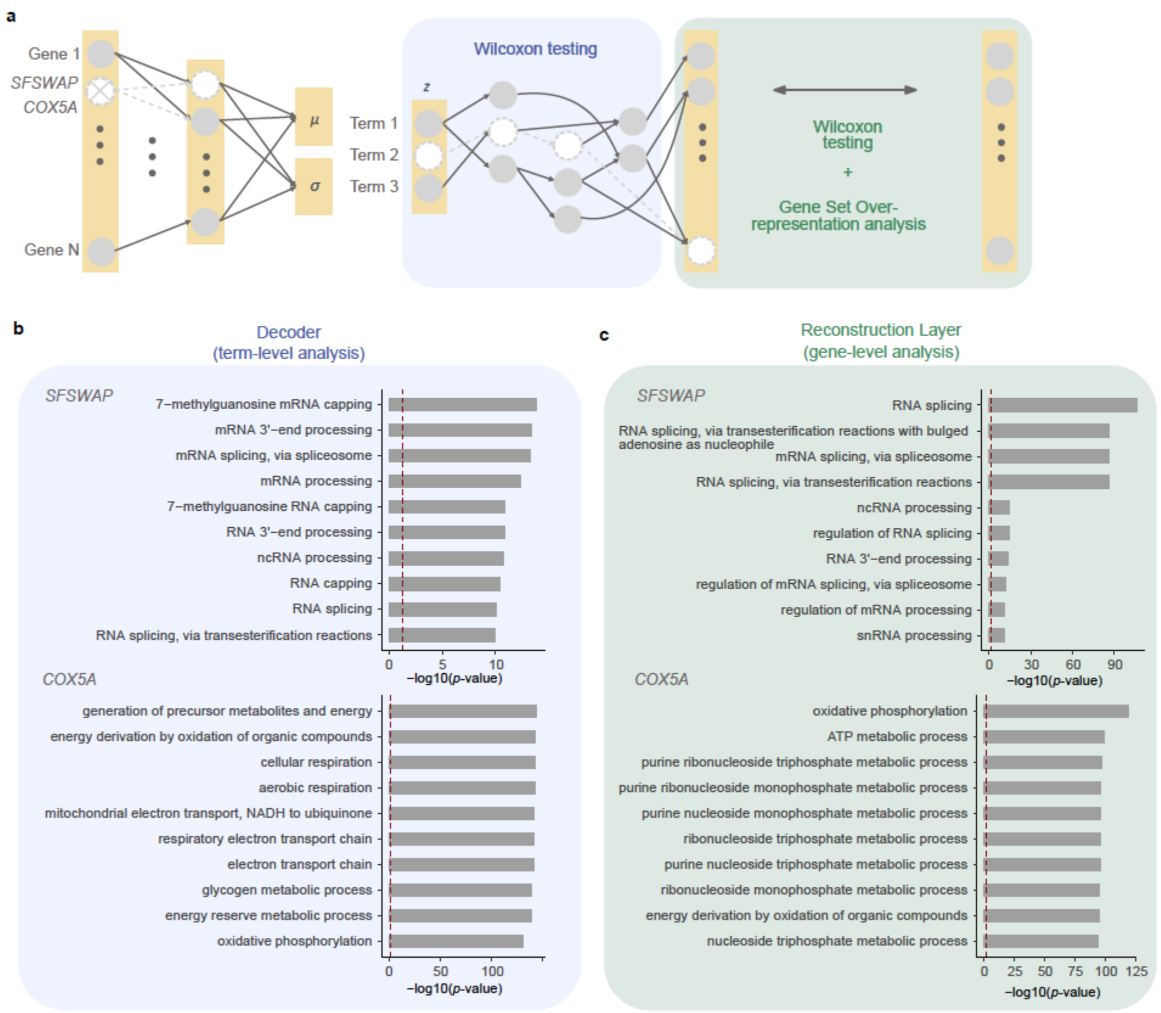

Fig. S5 OntoVAE can predict phenotypic outcome of a gene knockout (here: *SFSWAP* and *COX5A*). a, b, c as in Fig. 3a. **b** Barplots displaying the term-level analysis results for *SFSWAP* and *COX5A*. **c** Barplots displaying the gene-level analysis results for *SFSWAP* and *COX5A*.

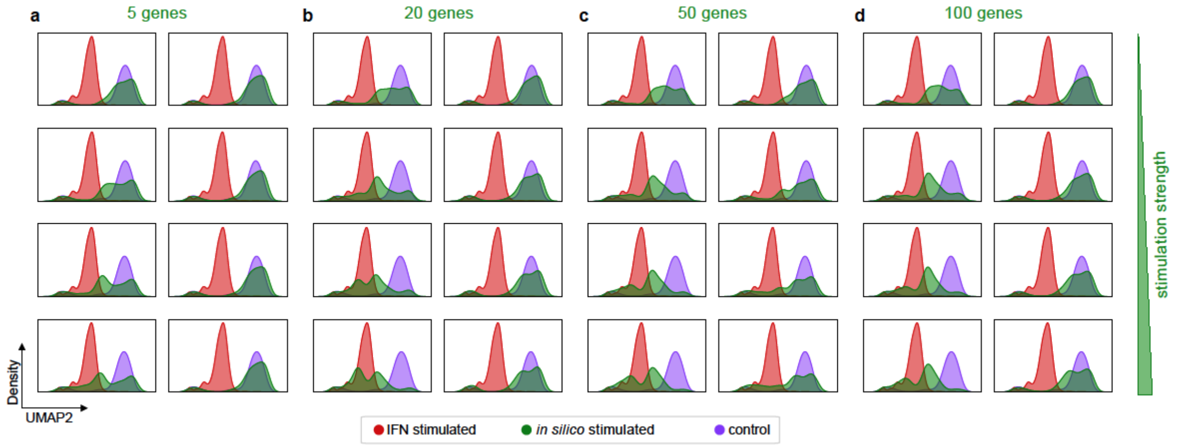

Fig. S6 *In silico* stimulated CD4T cells approach ground truth IFN-treated CD4T cells in latent space embedding. GO pathway activities for control CD4T cells (purple), IFN stimulated CD4T cells (red), and control CD4T cells that had been stimulated *in silico* (green) were obtained by running the samples through the OntoVAE model that had been trained on control data only. UMAP was computed on control and IFN stimulated cells, and *in silico* stimulated cells were projected into the UMAP space. Density plots are showing the distribution of cells over UMAP2, with stimulation strength increasing from top to bottom. For the stimulation, values of 2, 4, 6, and 8 were added to cells with non-zero values for the respective gene. The shift in cells is displayed for the stimulation of 5 genes (**a**), 20 genes (**b**), 50 genes (**c**), and 100 genes (**d**), with the left panels showing stimulation of the top n genes, and the right panels showing stimulation of random n genes from the leading edge.

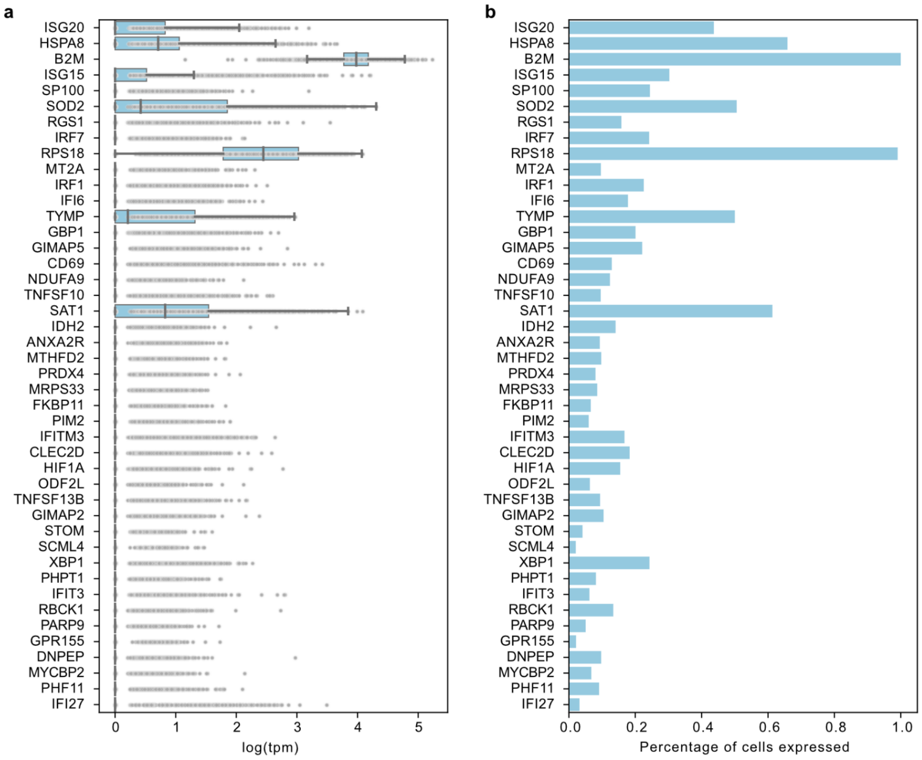

Fig. S7 OntoVAE can predict genes with low expression in training data. Displayed genes are found in the intersection of genes upregulated upon IFN-ß treatment in CD4T cells and genes predicted by OntoVAE to influence the node *type I interferon signaling pathway*. **a** Boxplots show the expression of the 44 genes in the training data, **b** barplots show what percentage of cells in the training data expresses the gene. Note that the training data consists of all unstimulated cells from the PBMC dataset.

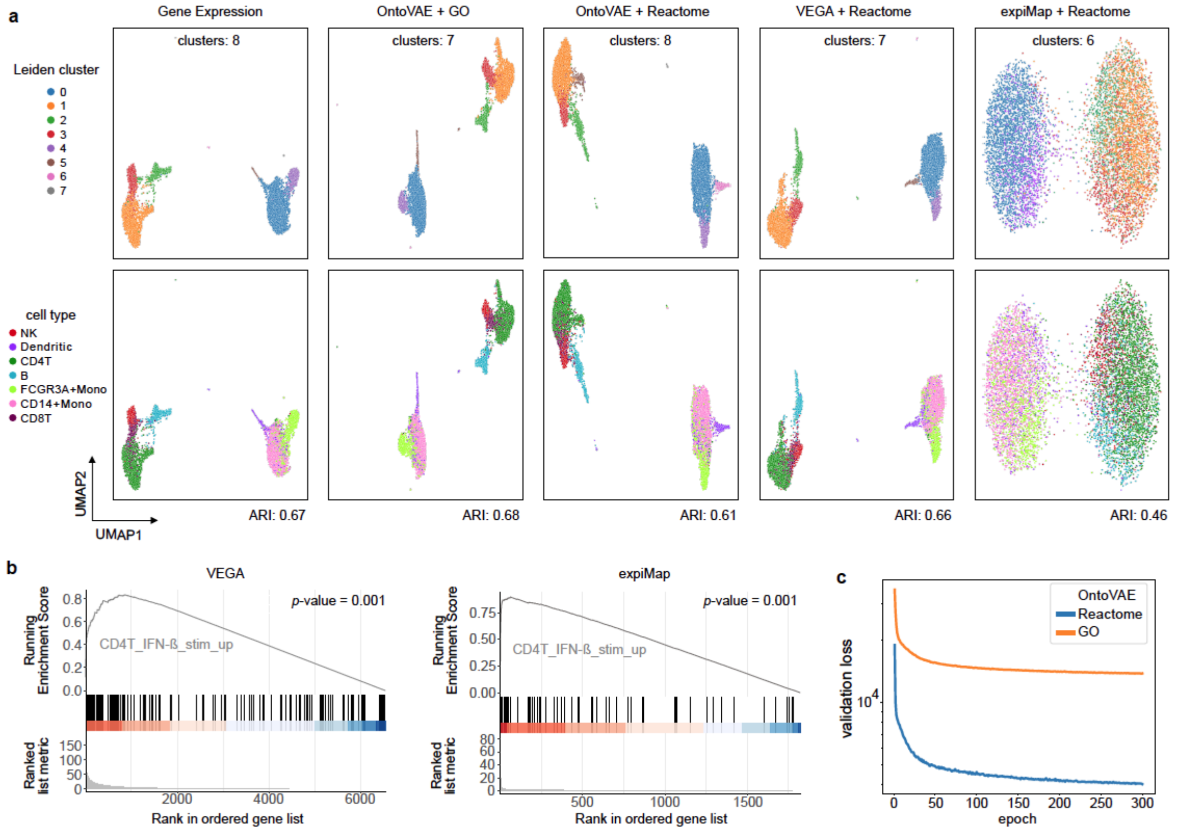

Fig. S8 Comparison of OntoVAE with other models. a Leiden clustering of unstimulated PBMC cells was performed directly on gene expression data, and on the latent space of OntoVAE + GO, OntoVAE + Reactome, VEGA + Reactome, and expiMap + Reactome, using resolutions of 0.2, 0.2, 0.4, 0.3, and 1, respectively. Adjusted rand index (ARI) was calculated for all five approaches. **b** The same analysis was carried out as in Figure 4, but this time, the one-layer decoder models VEGA (left panel) and expiMap (right panel) have been trained with Reactome pathways in the latent space. GSEA plots show the enrichment of CD4T_IFN-ß_stim_up in the list of genes ranked according to their influence on the Reactome_Interferon_Alpha_Beta_Signaling node. **c** Comparison of validation loss curves of OntoVAE trained with Reactome, and OntoVAE trained with GO.

	Depth	Layer	Input dim	Output dim	Connections
**Encoder**					
		Linear<br>BatchNorm<br>Dropout (p=0.2)<br>ReLU	19387	630 x n	
**Mu**					
(Latent space)	0	Linear<br>Dropout (p=0.5)	630 x n	630 x n	
**Logvar**					
(Latent space)	0	Linear<br>Dropout (p=0.5)	630 x n	630 x n	
**Decoder**					
	1	Linear	630 x n	14 x n	15 x n^2
	2	Linear	(630 + 14) x n	67 x n	71 x n^2
	3	Linear	(644 + 67) x n	218 x n	247 x n^2
	4	Linear	(711 + 218) x n	348 x n	471 x n^2
	5	Linear	(929 + 348) x n	484 x n	745 x n^2
	6	Linear	(1277 + 484) x n	510 x n	839 x n^2
	7	Linear	(1761 + 510) x n	451 x n	766 x n^2
	8	Linear	(2271 + 451) x n	270 x n	526 x n^2
	9	Linear	(2722 + 270) x n	143 x n	296 x n^2
	10	Linear	(2992 + 143) x n	71 x n	151 x n^2
	11	Linear	(3135 + 71) x n	30 x n	57 x n^2
	12	Linear	(3206 + 30) x n	7 x n	13 x n^2
	13	Linear	(3236 + 7) x n	2 x n	4 x n^2
(Reconstruction layer)	14	Linear	(3243 + 2) x n	19387	162,850 x n

Table S1 Structure of OntoVAE model with GO-decoder that was trained on GTEx bulk RNA-seq samples with trimming thresholds 1000 and 30. n = number of neurons per term. The model consists of a fully connected, non-linear encoder, which is coupled to a sparse, linear decoder. The latent space is implemented as two separate layers, mu and logvar. The *Depth* column indicates the depth layer of the ontology that is represented by this network layer, the *Layer* column shows the structure of each layer. *Input dim* and *Output dim* show how many features are used as input and output for the given layer. The dimensions are defined by the used and trimmed ontology, the number of genes that can be mapped to it, and the number of neurons used to model one term (n). Since layers are being concatenated at each step in the decoder, the *Input dim* for the current layer is always the sum of the previous layer and the current layer. Thus, the *Output dim* column also shows how many GO terms are belonging to each depth layer, for example 630 terms to layer 0, 14 terms to layer 1, and so on. The *Connections* column indicates how many connections are present in the binary mask connecting the two layers, for example 15 connections between layer 0 and layer 1, 71 connections between cat(layer 0, layer 1) and layer 2, and so on. The total number of connections is dependent of n. See also Suppelemtary Figure 2.

	Depth	Layer	Input dim	Output dim	Connections
**Encoder**					
		Linear<br>BatchNorm<br>Dropout (p=0.2)<br>ReLU	4774	703 x n	
**Mu**					
(Latent space)	0	Linear<br>Dropout (p=0.5)	703 x n	703 x n	
**Logvar**					
(Latent space)	0	Linear<br>Dropout (p=0.5)	703 x n	703 x n	
**Decoder**					
	1	Linear	703 x n	30 x n	$30 \times n^2$
	2	Linear	(703 + 30) x n	150 x n	$155 \times n^2$
	3	Linear	(733 + 150) x n	372 x n	$398 \times n^2$
	4	Linear	(883 + 372) x n	806 x n	$909 \times n^2$
	5	Linear	(1255 + 806) x n	829 x n	$998 \times n^2$
	6	Linear	(2061 + 829) x n	633 x n	$795 \times n^2$
	7	Linear	(2890 + 633) x n	428 x n	$559 \times n^2$
	8	Linear	(3523 + 428) x n	331 x n	$461 \times n^2$
	9	Linear	(3951 + 331) x n	161 x n	$231 \times n^2$
	10	Linear	(4282 + 161) x n	56 x n	$109 \times n^2$
	11	Linear	(4443 + 56) x n	20 x n	$30 \times n^2$
	12	Linear	(4499 + 20) x n	5 x n	$10 \times n^2$
(Reconstruction layer)	13	Linear	(4519 + 5) x n	4774	190,487 x n

Table S2 Structure of OntoVAE model with HPO-decoder that was trained on GTEx bulk RNA-seq samples with trimming thresholds 1000 and 10. n = number of neurons per term. For an explanation of the table, see the caption of Table S1.

	Depth	Layer	Input dim	Output dim	Connections
**Encoder**					
		Linear<br>BatchNorm<br>Dropout (p=0.2)<br>ReLU	19469	630 x n	
**Mu**					
(Latent space)	0	Linear<br>Dropout (p=0.5)	585 x n	585 x n	
**Logvar**					
(Latent space)	0	Linear<br>Dropout (p=0.5)	585 x n	585 x n	
**Decoder**					
	1	Linear	585 x n	14 x n	15 x n^2
	2	Linear	(585 + 14) x n	71 x n	76 x n^2
	3	Linear	(599 + 71) x n	207 x n	233 x n^2
	4	Linear	(670 + 207) x n	360 x n	495 x n^2
	5	Linear	(877 + 360) x n	467 x n	732 x n^2
	6	Linear	(1237 + 467) x n	479 x n	795 x n^2
	7	Linear	(1704 + 479) x n	423 x n	734 x n^2
	8	Linear	(2183 + 423) x n	242 x n	473 x n^2
	9	Linear	(2606 + 242) x n	133 x n	278 x n^2
	10	Linear	(2848 + 133) x n	66 x n	143 x n^2
	11	Linear	(2981 + 66) x n	28 x n	54 x n^2
	12	Linear	(3047 + 28) x n	6 x n	12 x n^2
	13	Linear	(3075 + 6) x n	2 x n	4 x n^2
(Reconstruction layer)	14	Linear	(3081 + 2) x n	19469	151,495 x n

Table S3 Structure of OntoVAE model with GO-decoder that was trained on Kang PBMC scRNA-seq data with trimming thresholds 1000 and 30. n = number of neurons per term. For an explanation of the table, see the caption of Table S1.
